# IGFBPL1 Regulates Axon Growth through IGF-1-mediated Signaling Cascades

**DOI:** 10.1038/s41598-018-20463-5

**Published:** 2018-02-01

**Authors:** Chenying Guo, Kin-Sang Cho, Yingqian Li, Kissauo Tchedre, Christian Antolik, Jie Ma, Justin Chew, Tor Paaske Utheim, Xizhong A. Huang, Honghua Yu, Muhammad Taimur A. Malik, Nada Anzak, Dong Feng Chen

**Affiliations:** 1 Schepens Eye Research Institute, Massachusetts Eye and Ear, Department of Ophthalmology, Harvard Medical School, Boston, MA 02114 USA; 2 Pritzker School of Medicine, Biological Sciences Division, University of Chicago, Chicago, IL 60637 USA; 3 Department of Medical Biochemistry, Oslo University Hospital, Kirkeveien 166, 0407 Oslo, Norway; 4 Oncology Translational Medicine, Novartis Institutes for BioMedical Research, Inc., Cambridge, MA 02138 USA; 5 Guys, Kings & St Thomas’ School of Medicine, Hodgkin Building, Guy’s Campus, King’s College London, London, UK; 6 Boston VA Healthcare System, 150 S. Huntington Ave, Boston, MA 02130 USA

## Abstract

Activation of axonal growth program is a critical step in successful optic nerve regeneration following injury. Yet the molecular mechanisms that orchestrate this developmental transition are not fully understood. Here we identified a novel regulator, insulin-like growth factor binding protein-like 1 (IGFBPL1), for the growth of retinal ganglion cell (RGC) axons. Expression of IGFBPL1 correlates with RGC axon growth in development, and acute knockdown of IGFBPL1 with shRNA or IGFBPL1 knockout *in vivo* impaired RGC axon growth. In contrast, administration of IGFBPL1 promoted axon growth. Moreover, IGFBPL1 bound to insulin-like growth factor 1 (IGF-1) and subsequently induced calcium signaling and mammalian target of rapamycin (mTOR) phosphorylation to stimulate axon elongation. Blockage of IGF-1 signaling abolished IGFBPL1-mediated axon growth, and *vice versa*, IGF-1 required the presence of IGFBPL1 to promote RGC axon growth. These data reveal a novel element in the control of RGC axon growth and suggest an unknown signaling loop in the regulation of the pleiotropic functions of IGF-1. They suggest new therapeutic target for promoting optic nerve and axon regeneration and repair of the central nervous system.

## Introduction

The exuberant growth of axons in the mammalian central nervous system (CNS) becomes markedly reduced as neurons mature. This is in part a result of the developmental shutdown of the axon growth program which contributes critically to the failure of CNS regeneration and repair after injury^1–4^. Retinal ganglion cells (RGCs), which have long served as a standard model of CNS neurons, switch off the intrinsic axon growth program during the prenatal period in mice^5–7^. Upon differentiation, developing RGCs must receive accurate and timely stimulation to initiate the intrinsic axon growth program for successful development of the optic nerve. One potential mechanism is governed through secretory factors that, when bound to neurons, switch on intracellular axon growth cascades, while their absence turns off the axon growth signals and leads to loss of nerve regenerative capacity. These factors may represent important targets for therapeutic interventions to promote regeneration after CNS injury. To date, the molecular signals that orchestrate the transition of RGC axon growth cascades have not been fully understood.

Studies have suggested that IGF-1 (insulin-like growth factor-1) is required for the growth of CNS axons. The question remains as how IGF-1 activates the axon growth machinery in the developing, but not adult, neurons of the CNS. Intriguingly, IGF-1 mediates not only axon growth, but multiple other biological processes of developing neurons, including proliferation, survival, and synaptogenesis. To date, at least 7 IGF binding proteins (IGFBPs) and an IGFBP like protein 1 (IGFBPL1) are identified. We hypothesized that IGF-1 requires IGFBP, which may be expressed during certain period in development, to selectively enable the activation of IGF-1-induced pleiotropic signaling cascades. In the present study, we showed that IGFBPL1 is a critical co-factor of IGF-1 for its activation of axon growth cascades. Acute knockdown of IGFBPL1 with shRNA nullifies IGF-1-induced axon growth activities in developing RGCs. In contrast, administration of IGFBPL1 stimulated RGC axon elongation. IGFBPL1 activates the axonal growth machinery by binding with IGF-1 to enable intracellular Ca^2+^ elevation essential for axon extension. Finding of the function of IGFBPL1 in IGF-1-mediated axon growth function unveils a new regulatory element in its pleiotropic activities. IGFBPL1, as a secretory factor that directly regulates axonal growth, may present a possibility for pharmacological manipulation to promote axon regeneration and reverse vision loss after injury in human patients.

## Results

### Developmental loss of IGFBPL1 expression correlates with the cessation of RGC axon growth

**Figure 1:**
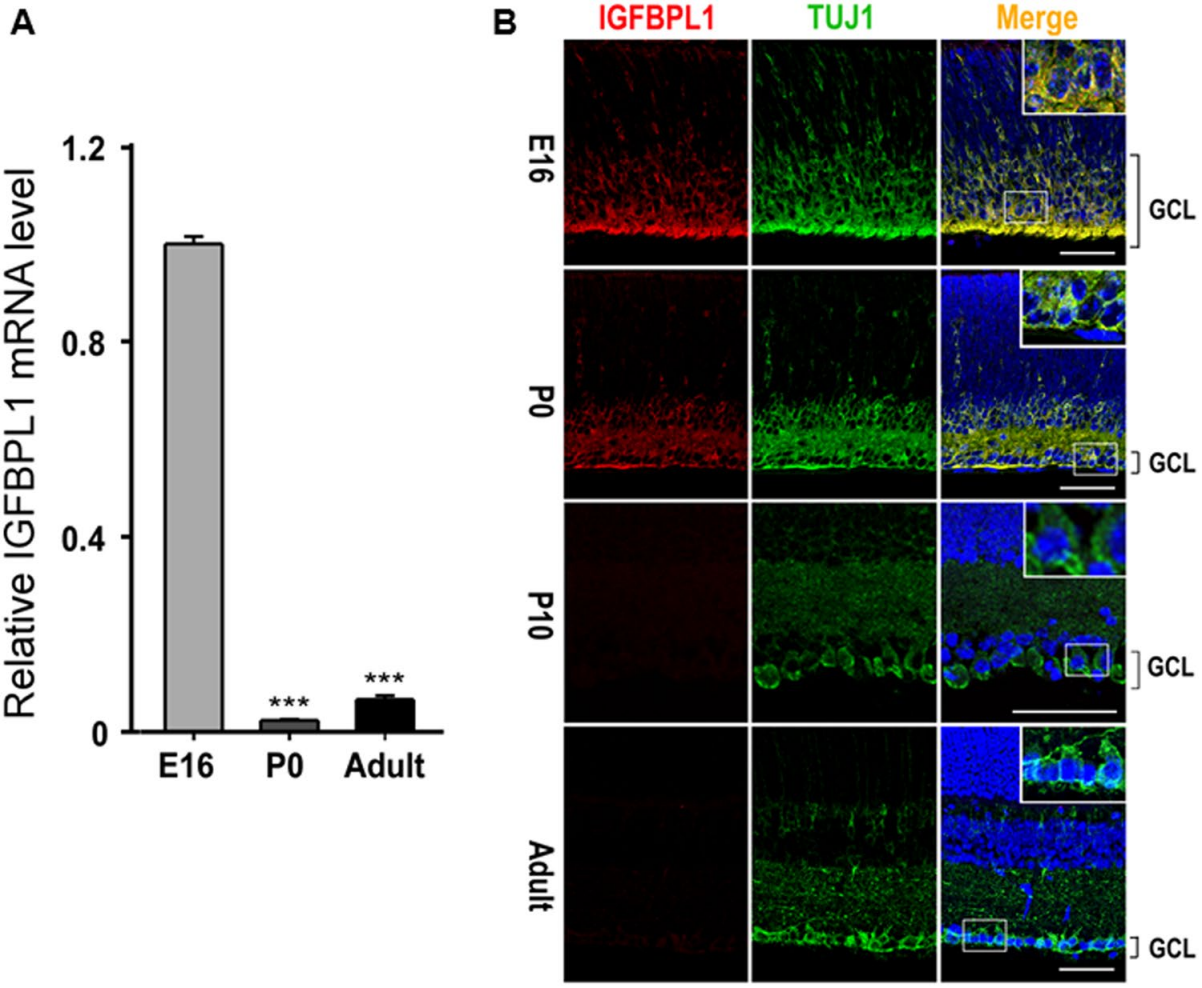
Developmental downregulation of IGFBPL1 in the retina. (**A**) Quantification of IGFBPL1 mRNA levels with qPCR (normalized to the E16 level) in the developing mouse retina showing drastic downregulation of IGFBPL1 expression at P0 (n = 5 mice/time point). ***P < 0.001, as compared to the E16 group by two-tailed student *t*-test. Error bars indicate standard error of mean (SEM). (**B**) Epifluorescence photomicrographs of retinal sections from E16, P0, P10 and adult mice (2 months) double-immunolabeled with primary antibodies against IGFBPL1 (red) and RGC specific marker Tuj1 (green); retinal sections were counterstained with a nuclear marker DAPI (blue). Note the bright labeling of IGFBPL1 in E16 ganglion cell layer (GCL) that was down-regulated after birth and became extinct after P10. GCL, ganglion cell layer. Scale bars: 50 µm.

Mouse RGC axons switch off exuberant growth between embryonic day 16 (E16) and birth (P0)^6,8^ presents a unique tractable model for identification of candidate regulators of axonal growth by comparing gene expression profiles. Through analysis of cDNA microarray data obtained from E16 and P0 retinas, a novel secretory protein IGFBPL1 emerged as a candidate molecule whose levels of expression correlated with RGC axon growth capacity. This result was confirmed by quantitative RT-PCR (qPCR) that examined RNA levels in the retinas of E16, P0, and adult mice (Fig. 1A). β-III-tubulin, as shown by our lab and others, is a marker of RGCs in the GCL of the mouse retina^9,10^. Double-immunolabeling of IGFBPL1 and a primary antibody against β-III-tubulin (Tuj1; Fig. 1B and Supplementary Fig. 1) revealed that IGFBPL1 was highly enriched in the ganglion cell layer (GCL) of E16 retinas. Its expression dropped sharply at P0 and became non-detectable after P10 to adulthood (Fig. 1A,B). During development, exuberant growth of RGC axons is largely reduced by P0, while a sub-cohort of late-born RGCs is reported to continue extending axons up to P6-7^11–13^. The spatiotemporal expression of IGFBPL1 thus correlates precisely with the growth status of RGC axons, raising the possibility that IGFBPL1 participates in the regulation of RGC axonal growth.

### IGFBPL1 mediates RGC axon growth *in vitro*

**Figure 2:**
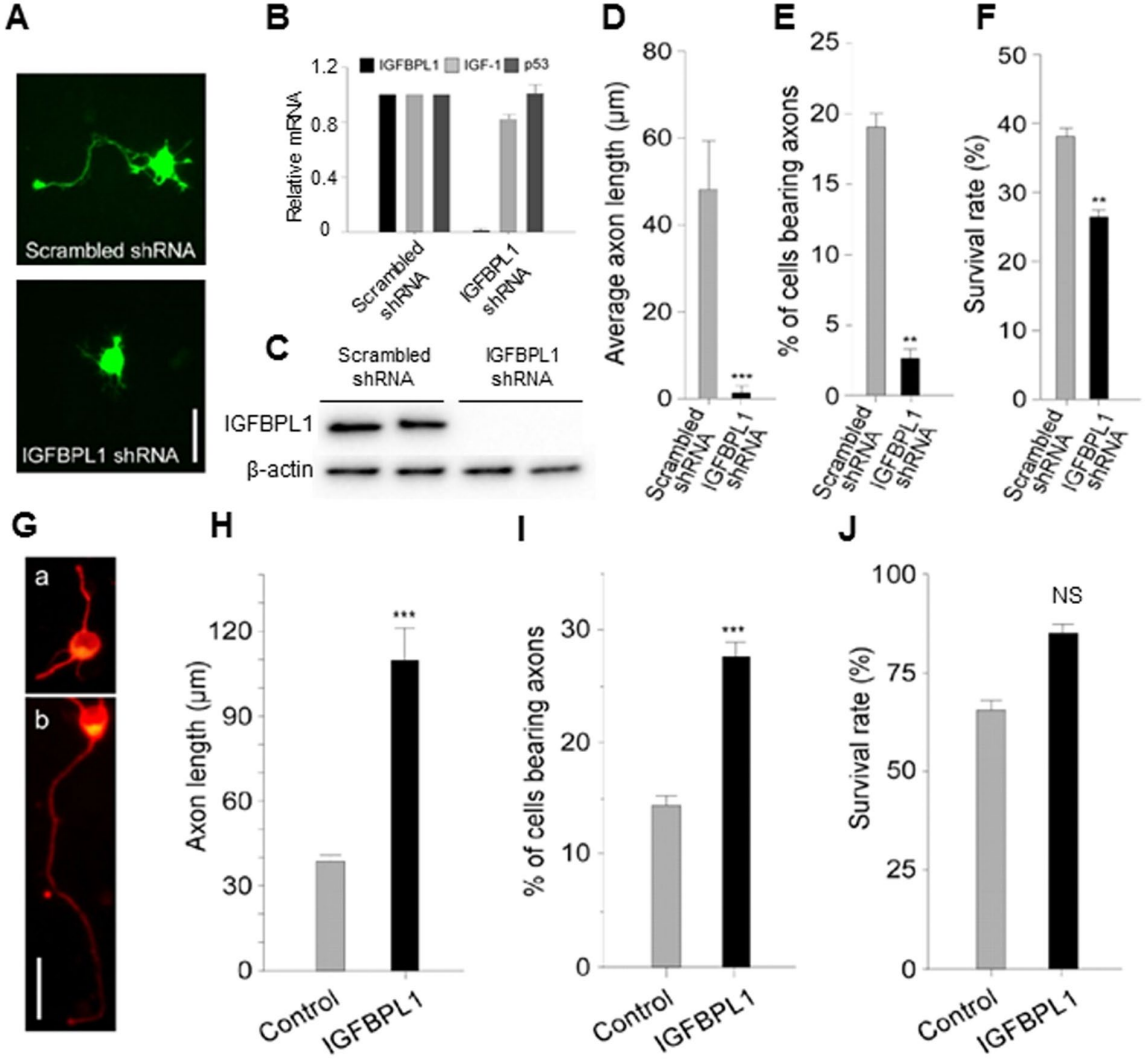
Role of IGFBPL1 for RGC axonal outgrowth *in vitro*. (**A**) Representative photomicrographs of cultured RGCs infected with lentiviral vectors carrying scrambled shRNA or IGFBPL1 shRNA tagged by a GFP reporter gene. Scale bar: 20 um. (**B**) qPCR results of IGFBPL1, IGF-1 and p53 mRNA levels in P0 RGC cultures infected by lentiviruses carrying either scrambled or IGFBPL1 shRNAs. mRNA levels were normalized to that of the untreated RGCs. (**C**) Representative images of Western blot analysis of IGFBPL1 (upper) quantification in P0 RGC cultures infected by scrambled or IGFBPL1 shRNA**;** β-actin (lower) was used as a loading control. The original images are shown in Supplementary Fig. 2. (**D**) Quantification of axon length in P0 RGC cultures infected by lentiviruses carrying either scrambled or IGFBPL1 shRNAs. (**E,F**) Percentage of cells bearing axons (**E**) and cell survival (**F**) in cultures infected by lentiviral vectors carrying scrambled shRNA or IGFBPL1 shRNA. (**G**) Representative epifluorescence photomicrographs of cultured P0 RGCs in the absence (a) or presence (b) of IGFBPL1 (400 ng/ml). Cells were immunolabeled with Tuj1 (red). Scale bar: 20 um. (**H**–**J**) Quantification of axon length (**H**), percentage of cells bearing axons (**I**) and percentage of surviving cells (**J**) in cultured P0 RGCs treated with various concentrations of IGFBPL1, n = 6 cultures/group. ***P < 0.001, **P < 0.01, as compared to the control group by two-tailed student t-test. Error bars indicate standard error of mean (SEM).

To explore the role of IGFBPL1 in RGC axonal growth, we utilized shRNA silencing to acutely knockdown the expression of IGFBPL1 in a standard model of purified RGC cultures. Primary RGCs isolated from newborn (P0) mouse pups were infected with lentiviral particles carrying IGFBPL1 shRNA or scrambled shRNA tagged with green fluorescence protein (GFP). After 3 days of incubation, RGCs infected by IGFBPL1 shRNA, but not scrambled shRNA, exhibited a complete knockdown of IGFBPL1 mRNA and protein expression as revealed by qPCR and Western blot (Fig. 2A–C and Supplementary Fig. 2); the expression of other genes, e.g. IGF-1 and p53, was not affected. Accompanied with the knockdown of IGFBPL1, we noted an over 30-fold reduction in average axon length (Fig. 2D) and a 7-fold reduction in the number of RGCs bearing axons (Fig. 2E), but only a moderate reduction (~30%) of RGC survival, compared to cultures treated with scrambled shRNA (Fig. 2F). Notably, surviving RGCs in IGFBPL1 shRNA treated cultures did not grow neurites, indicating their impaired ability to grow axons. To test if IGFBPL1 can stimulate RGC axon outgrowth in culture, recombinant mouse IGFBPL1 protein was added to the cultured RGCs purified from P0 mouse pups (Fig. 2G). After 3 days of incubation, addition of IGFBPL1 significantly enhanced RGC axonal outgrowth (Fig. 2H,I). At the optimal concentration (400 ng/ml), administration of IGFBPL1 induced a 3-fold increase in axon length and 2-fold increase in the number of RGCs bearing axons; whereas, addition of IGFBPL1 did not significantly increase RGC survival (Fig. 2I). Thus, IGFBPL1 regulates RGC axonal growth without significantly affecting cell survival.

To determine if the axon growth promoting effect of IGFBPL1 is limited to RGCs, we knocked down IGFBPL1 in PC12 cells and mouse hippocampus neurons. Consistently, we observed reduced neurite length without significant change in cell survival in both PC12 (Supplementary Fig. 3A,B) and hippocampal neuron cultures (Supplementary Fig. 3C,D). Addition of IGFBPL1, in contrast, led to significantly increased neurite outgrowth in PC12 cell (Supplementary Fig. 4A,B) and hippocampal neuron cultures (Supplementary Fig. 4C,D). Together, these data indicate that IGFBPL1 is generally involved in the regulation of neuron axon growth.

### IGFBPL1 deficiency impaired RGC axon growth *in vivo*

**Figure 3:**
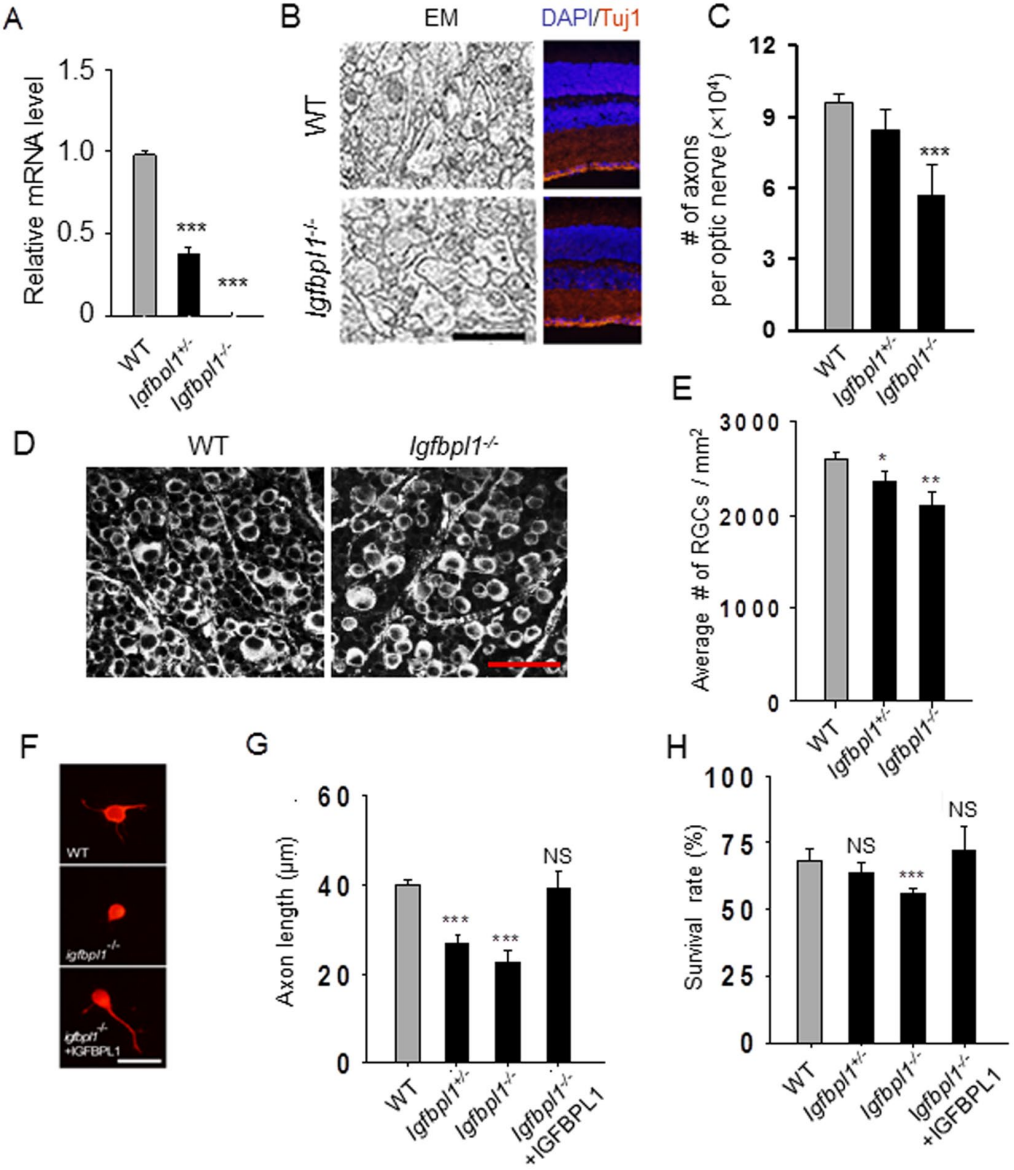
Impaired RGC axon development in *Igfbpl1*^−/−^ mice. (**A**) Quantification of IGFBPL1 mRNA levels in WT, *Igfbpl*^*+/−*^, and *Igfbpl1*^−/−^ mouse retinas with qPCR (n = 5 mice/group). (**B**) Representative images of RGC axon morphology in optic nerve cross sections and retinal laminar structure in P0 WT and *Igfbpl1*^−/−^ mice. Optic nerve sections were stained by paraphenylene diamine (PPD) to reveal myelinated axons (Scale bar: 5 µm). Retinal sections were immunolabeled with primary antibody against RGC specific marker Tuj1 (red) and counter-stained by a nuclear marker DAPI. No apparent differences in retinal laminar structure or cell densities were noted in WT and *igfbpl1*^−/−^ mice. Scale bar: 50 µm. (**C**) Axon counts in optic nerve sections of P0 WT, *Igfbpl1*^*+/−*^ and *Igfbpl1*^−/−^ mice (n > 5). (**D,E**) Representative images (**D**) and counts (**E**) of RGCs in Tuj1-immunolabeled retinal whole-mounts from adult WT and *Igfbpl1*^−/−^ mice. Scale bar: 50 µm. (**F**) Representative epifluorescence photomicrographs of RGCs from littermate P0 WT and *Igfbpl1*^−/−^ (KO) mouse pups cultured in the absence (middle panel) or presence (bottom panel) of IGFBPL1 protein and immunolabeled with Tuj1 (red). Addition of IGFBPL1 (200 ng/ml) rescued the neurite growth defect of *Igfbpl1*^−/−^ (KO) RGCs. Scale bar: 20 µm. (**G**,**H**). Quantification of RGC axon length (**G**) and cell survival (**H**) (n = 5/group). ***P < 0.001, **P < 0.01, *P < 0.05, as compared to the WT group by two-tailed student t-test. Error bars indicate standard error of mean (SEM).

To investigate if IGFBPL1 is required for RGC axonal growth *in vivo* during development, we generated *Igfbpl1* null mutant (*Igfbpl1*^−/−^) mice. Quantification with qPCR confirmed the absence of IGFBPL1 mRNA in homozygous knockout (*Igfbpl1*^−/−^) mice (Fig. 3A). *Igfbpl1*^−/−^ mice were viable and fertile without apparent growth defects or phenotypes. The retinas of newborn *Igfbpl1*^*+/−*^ and *Igfbpl1*^−/−^ mice displayed normal laminar structure, and immunohistochemistry. Results of qPCR that examined the expression of various retinal cell markers revealed no significant defects associated with photoreceptor and interneuron gene expression (not shown). In agreement with the specific localization of IGFBPL1 in RGCs, new born *Igfbpl1*^−/−^ mice exhibited a largely reduced number of axons (~40%) in the optic nerve compared to wild-type (WT) littermates (Fig. 3B,C), while they showed approximately 20% reduction in number of RGCs as compared to the WT littermates (Fig. 3D,E).

To verify that impaired axon growth in *Igfbpl1*^−/−^ mice is a direct consequence of IGFBPL1 deficiency, we isolated RGCs from newborn (P0) *Igfbpl1*^*+/−*^ and *Igfbpl1*^−/−^ mice. RGCs taken from P0 *Igfbpl1*^*+/−*^ and *Igfbpl1*^−/−^ mouse pups exhibited a 50% reduction in axon length in culture compared to WT littermates (Fig. 3F,G); whereas, the survival of RGCs of *Igfbpl1*^−/−^ mice was reduced by only ~15% compared to WT mice (Fig. 3H). Addition of IGFBPL1 protein to RGCs of *Igfbpl1*^−/−^ mice in culture completely rescued the axonal growth and survival defects (Fig. 3G,H), suggesting that these growth defects were a result of IGFBPL1 deficiency rather than developmental defects of other cellular cascades. The data supports that IGFBPL1 plays an important role in mediating RGC axon growth during development *in vivo*. The relatively milder impact of gene deletion of IGFBPL1 on RGC axon growth than the acute knockdown of IGFBPL1 with shRNA suggests possible functional redundancy among IGFBPs.

### IGFBPL1 regulates axon growth through binding to IGF-1

**Figure 4:**
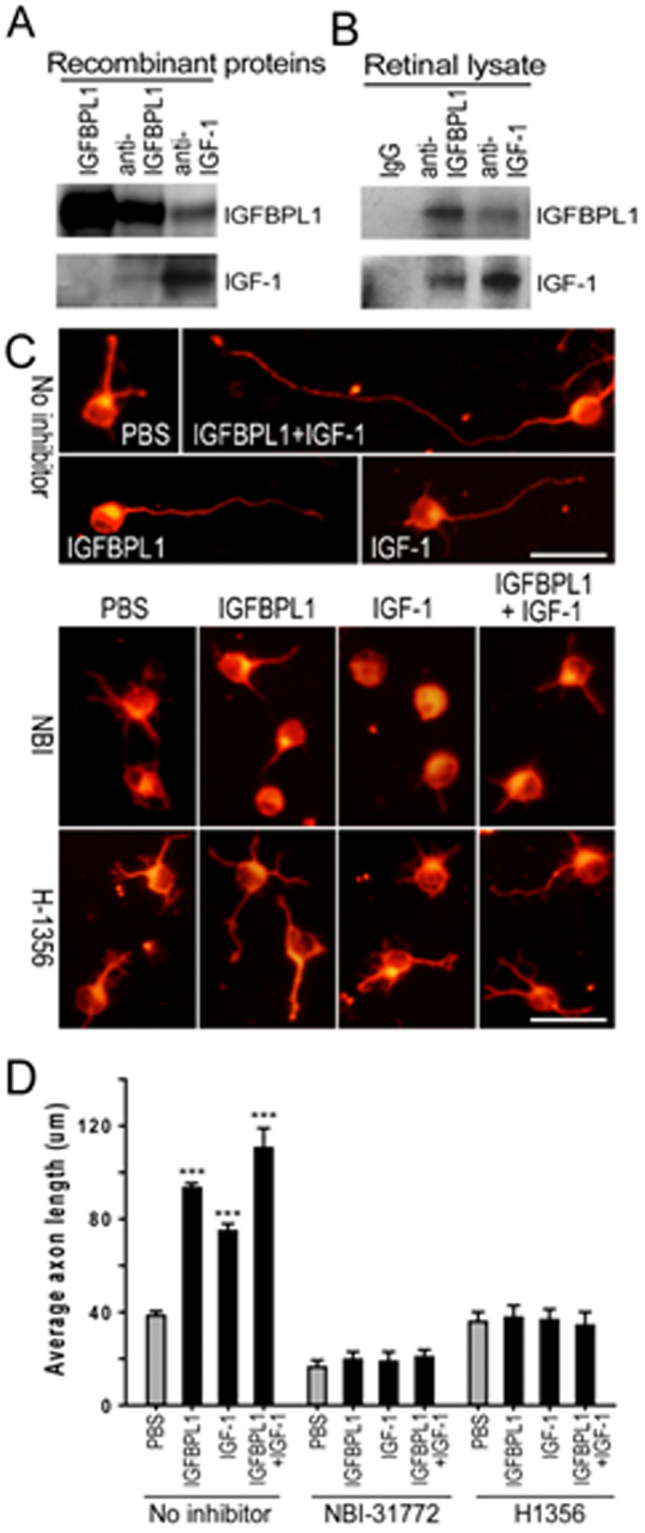
Function of IGF-1/IGF-1R signaling for IGFBPL1-mediated growth. (**A**) Co-IP of recombinant IGFBPL1 and IGF-1 proteins *ex vivo*. Anti-IGFBPL1 detected the presence of IGF-1 in the co-IP product and vice versa, anti-IGF-1 detected the presence of IGFBPL1 in the protein complex. (**B**) Co-IP detected IGFBPL1 (upper panel) and IGF-1 (lower panel) in protein complexes pulled down with primary antibodies against IGFBPL1 (middle) or IGF-1 (right). Left lane represents control co-IP result using a control rabbit IgG antibody. (**C**) Representative photomicrographs of cultured P0 RGCs treated with IGF-1 and/or IGFBPL1 in the absence or presence of NBI-31772 (NBI; 20 μM) or H1356 (40 μg/ml). Cells were immunolabeled with Tuj1. Scale bar: 20 μm. (**D**) Quantification of axon length in cultured RGCs (n = 5 cultures/group). Note that application of NBI-31772 or H1356 completely blocked either IGF-1- or IGFBPL1-induced axonal outgrowth. ***P < 0.001 as compared to the control (PBS) group by two-tailed student t-test. Error bars indicate standard error of mean (SEM).

Because IGFBPL1 shares an IGF-1 binding domain with all IGFBPs, we asked if IGFBPL1 binds IGF-1 in axon growth regulation. We examined the direct physical interaction of IGFBPL1 and IGF-1 using purified recombinant proteins. Bacterially produced IGF-1 was co-immunoprecipitated (co-IPed) with recombinant IGFBPL1, which was purified from a mouse myeloma cell line. Notably, recombinant IGF-1 bound to IGFBPL1 *ex vivo* (Fig. 4A), suggesting that IGF-1 can directly bind with IGFBPL1. To test if IGFBPL1 directly interacts with IGF-1 *in vivo* under physiological conditions, retinal lysates of newborn mice were co-IPed using IGFBPL1 or IGF-1 antibodies. IGFBPL1 was precipitated from the retinal lysates with an anti-IGF-1 antibody, and reciprocally, IGF-1 was detected in the co-IPed product pulled down by anti-IGFBPL1 antibody (Fig. 4B). These results indicate the physical interactions of IGFBPL1 and IGF-1 *in vivo* at the physiologically relevant protein levels.

We proposed that the interaction between IGF-1 and IGFBPL1 enables RGC axonal growth signaling. To test this, NBI-31772 (NBI), a non-peptide ligand that displaces IGF-1 from all currently known IGF-1 binding proteins^14^, was administered to P0 RGC cultures. NBI completely abolished the axon growth-promoting effect of IGFBPL1 (Fig. 4C,D). Moreover, H1356, an IGF-1 analog that competitively blocks the binding of IGF-1 with its receptor IGF-1R, also abolished the axonal growth promoting activity of IGFBPL1 (Fig. 4C,D). Thus, IGFBPL1 requires physical interaction with IGF-1 and activation of IGF-1R to promote RGC axon growth.

### IGFBPL1 is a co-factor for IGF-1 in the control of axonal growth during development

**Figure 5:**
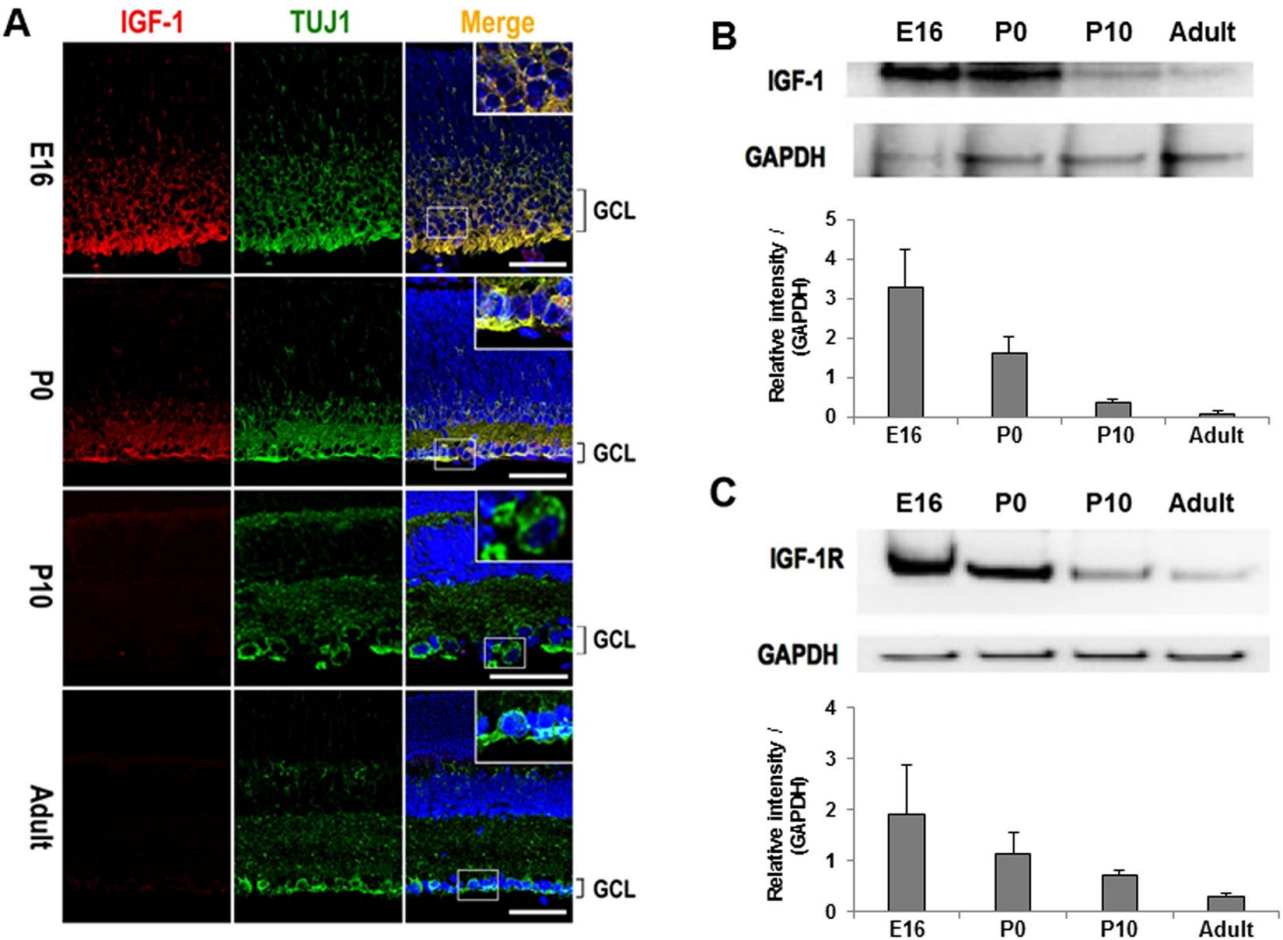
Expression of IGF-1 and IGF-1R in the developing retina. (**A**) Epifluorescence photomicrographs of retinal sections taken from E16, P0, P10 and adult mice double-immunolabeled with primary antibodies against IGF-1 (red; left panel) and Tuj1 (green; middle panel) and counterstained with nuclear marker DAPI (blue). GCL, ganglion cell layer. Scale bars: 50 µm. (**B**,**C**) Western blot quantification of IGF-1 (**B**) and IGF-1R (**C**) expression in retinal lysates taken from E16, P0, P10 and adult mice. Western blot quantifications were normalized to GAPDH and reported as relative intensity compared to GAPDH in the bar charts.

We proposed that if presence of both IGFBPL1 and IGF-1 are needed for stimulating RGC axon growth, IGF-1 and IGF-1R should co-exist with IGFBPL1 in the developing retina when RGCs undergo axon elongation. Results of immunohistochemistry and Western blot confirmed that IGF-1 was highly enriched in the ganglion cell layer of the E16 retina, and its level of expression persisted in P0 RGCs but was largely diminished after P10 (Fig. 5A,B). This pattern of IGF-1 expression parallels that of IGF-1R in the retina (Fig. 5C) and is in agreement with the reported expression profile of IGF-1^15^. Thus, IGF-1, IGF-1R and IGFBPL1 are concurrently expressed in the perinatal retina when RGCs exuberantly grow axons. Down-regulation of IGFBPL1, and to a less extent for IGF-1 or IGF-1R, correlates with the cessation of RGC axon elongation in development.

**Figure 6:**
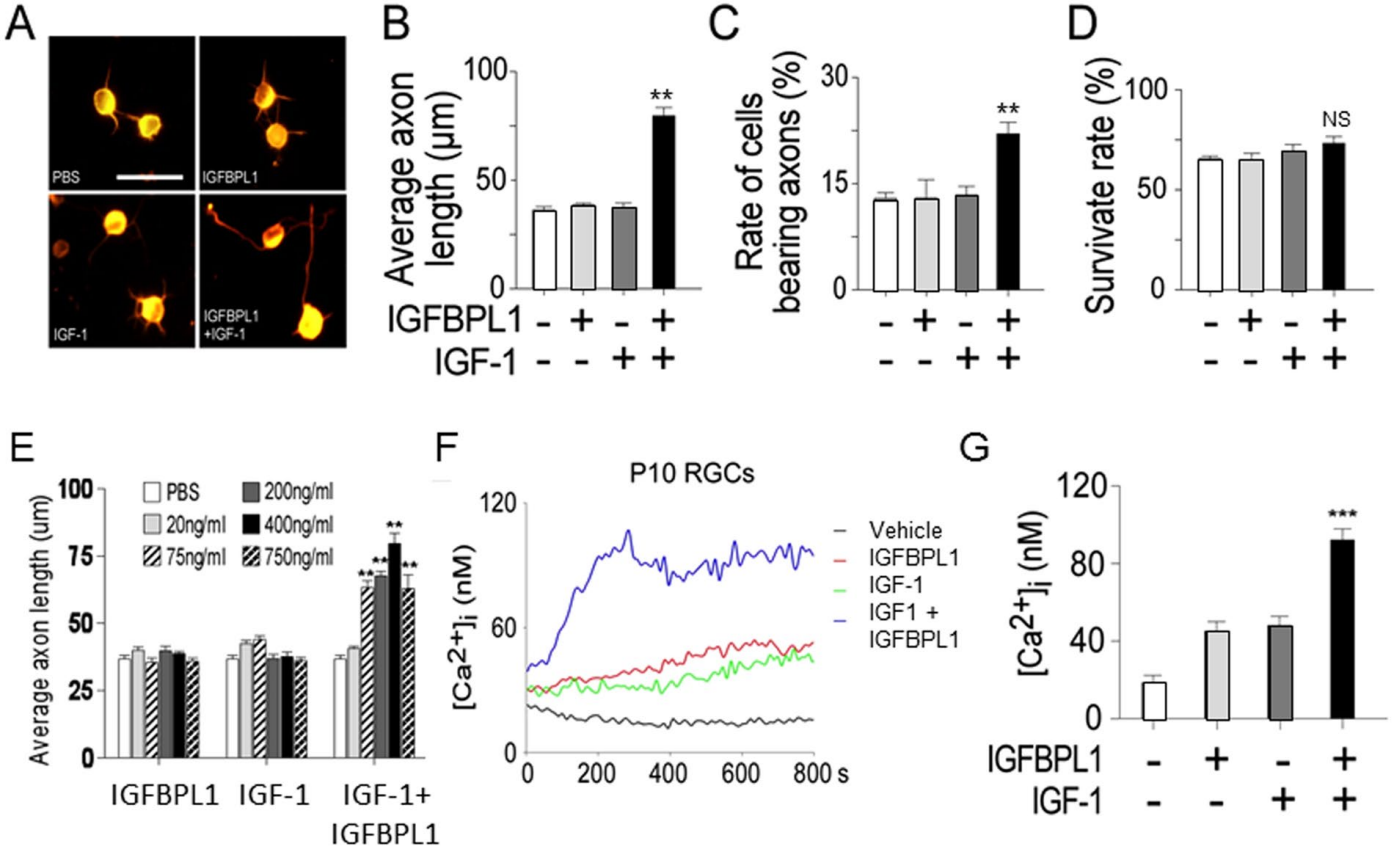
Activation of Ca^2+^ signaling by co-application of IGFBPL1 and IGF-1. (**A**) Representative photomicrographs of cultured RGCs derived from P10 mouse pups immunolabeled with Tuj1 to reveal RGCs and axonal processes. Cells were treated with IGF-1, IGFBPL1 or both for 3 days, and cells treated with PBS served as vehicle controls. Scale bar: 20 μm. (**B**,**C**) Quantification of axon length (**B**) and percentage of cells bearing axons (**C**) in cultured P10 RGCs. (**D**) Quantification of RGC survival (n = 5 cultures/group). (**E**) Quantification of axonal growth in cultured P10 RGCs treated by varying doses of IGFBPL1 and/or IGF-1. n = 5 cultures/group. **P < 0.01 and ***P < 0.001, as compared to the controls by two-tailed student t-test. Error bars indicate standard error of mean (SEM). (**F**) Changes of [Ca^2+^]i in cultured P10 RGCs following administration of IGFBPL1, IGF-1 or IGFBPL1 + IGF-1. Elevation of [Ca^2+^]i was induced only when both IGFBPL1 and IGF-1 were added. (**G**) Quantification of [Ca^2+^]i levels in different treatment groups at 800 s time point after stimulation (n = 5 cultures/group, and 10–20 neurons were recorded from each group).

To further investigate if IGFBPL1 functions conjointly with IGF-1 to induce axonal growth, we examined RGCs isolated from P10 (rather than P0) mouse pups, when the retina produces minimal amounts of endogenous IGF-1 (Fig. 5A,B) and IGFBPL1 (Fig. 1B). As expected, P10 RGCs exhibited minimal axonal growth when was treated with a vehicle control. Addition of IGF-1 or IGFBPL1 *alone*, at increasing doses up to 750 ng/ml, to P10 RGC cultures did not significantly promote axon growth when compared with the control group (Fig. 6A–E). In contrast, co-administration of IGF-1 and IGFBPL1, even at low concentrations (e.g., 75 ng/ml), induced significant axon growth through increased axon length and numbers without improvement of cell survival (Fig. 6A–E). This result strongly supports that IGFBPL1 is a co-factor of IGF-1 for activating the axon growth signaling.

### IGFBPL1 enables IGF-1-induced Ca^2+^ signaling to promote axonal growth

**Figure 7:**
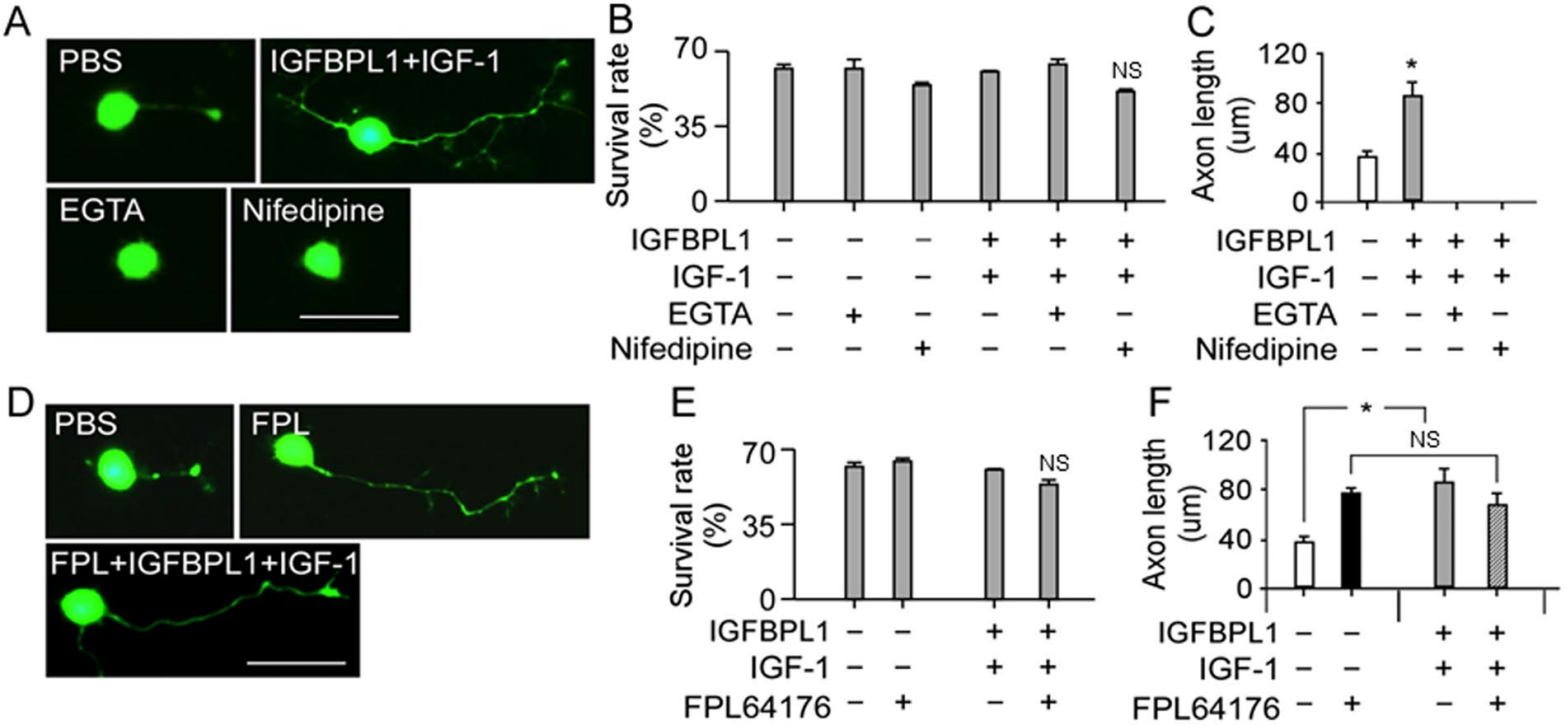
Requirement of intracellular Ca^2+^ elevation for IGFBPL1 and IGF-1-mediated RGC axonal growth. (**A**–**C**), Representative photomicrographs of RGC cultures (**A**), survival rate (**B**) and quantification of axon length (**C**) in cultures treated with PBS or IGFBPL1 + IGF-1 in the absence or presence of Ca^2+^ chelators EGTA or Ca^2+^ channel blocker Nifedipine. Cells and neuronal processes were visualized with Calcein AM. Scale bar: 20 µm. Addition of Ca^2+^ blockers completely abolished RGC axon outgrowth. (**D**–**F**) Representative photomicrographs (**D**), survival rate (**E**) and quantification of axon length (**F**) from cultured RGCs treated with PBS or IGFBPL1 + IGF-1 in the presence of a potent L-type Ca^2+^ channel activator FPL64176. Cells and neuronal processes were visualized with Calcein AM. Scale bar: 20 µm. *P < 0.05, as compared to vehicle control treated group by two-tailed student tests; NS: non-significant. Error bars indicate standard error of mean (SEM).

Elevation of intracellular calcium ([Ca^2+^]_i_) is an essential component of the signaling cascades required for the initiation of RGC axon growth^8^. We next examined the changes of [Ca^2+^]_i_ induced by IGFBPL1 and/or IGF-1 in P10 RGC cultures. Treatment with either IGFBPL1 or IGF-1 alone did not induce significant changes in [Ca^2+^]_i_ when compared to vehicle treated cultures (Fig. 6F,G), corresponding to their inability to stimulate axonal growth in P10 RGCs. In contrast, co-application of IGFBPL1 and IGF-1 induced robust elevation of [Ca^2+^]_i_ over the control group (Fig. 6F,G). To determine if this elevation of [Ca^2+^]_i_ was responsible for the axon growth-promoting activities, we blocked [Ca^2+^]_i_ change with Ca^2+^ chelators, EGTA (ethylene glycol-bis(β-aminoethyl ether)-N,N,N′,N′-tetraacetic acid)^16,17^ or L-type Ca^2+^ channel antagonist Nifedipine^18,19^. Administration of EGTA or Nifedipine blocked the axon growth-promoting effects induced by co-application of IGFBPL1 and IGF-1 (Fig. 7A,C) without affecting cell survival (Fig. 7B). In contrast, administration of Ca^2+^ channel agonist FPL64176 (FPL)—a potent L-type Ca^2+^ channel activator^20,21^—to P10 RGC cultures stimulated robust axonal outgrowth similar to what was seen in cultures receiving IGFBPL1 and IGF-1 co-treatment (Fig. 7D–F). FPL induced a larger than 2-fold increase in axon length over that of the control group. Moreover, addition of FPL to RGC cultures receiving IGFBPL1 and IGF-1 co-treatment did not further enhance their axon growth activity (Fig. 7D), suggesting that IGF/IGFBPL1 and FPL work through a common mechanism. Thus, the presence of IGFBPL1 enables IGF-1 to activate [Ca^2+^]_i_ signaling and the intracellular events that lead to axonal growth.

**Figure 8:**
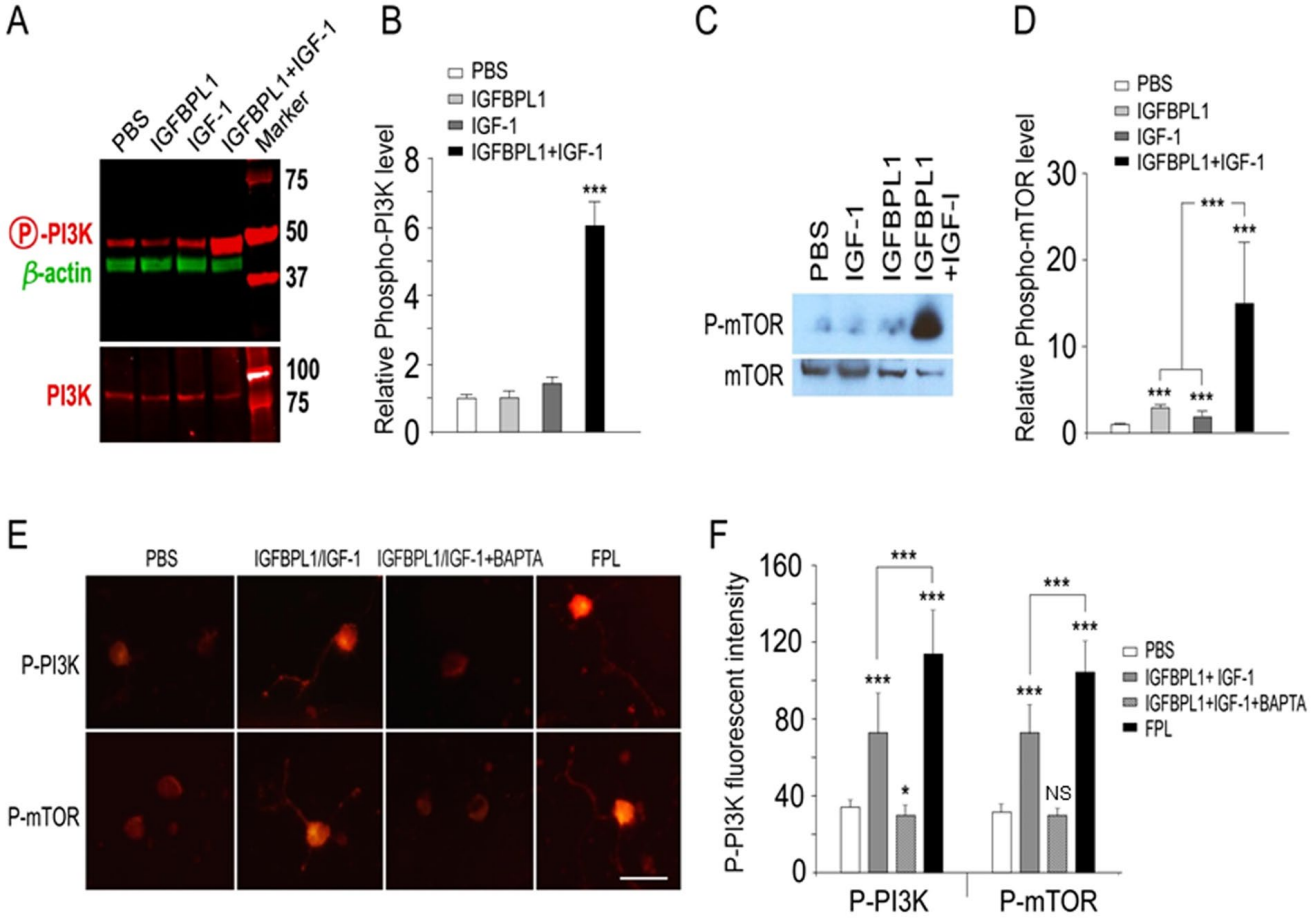
Activation of PI3K and mTOR signals by IGFBPL1 and IGF-1-mediated Ca^2+^ signaling. (**A**–**D**) Representative Western blots of triplicate experiments (**A**,**C**) and quantification (**B**,**D**) of PI3K and mTOR phosphorylation following treatment with IGFBPL1, IGF-1, or both in P10 retinal cultures. The levels of phosphorylated PI3K (P-PI3K) and phosphorylated mTOR (P-mTOR) increased drastically in cultures co-treated with IGFBPL1 and IGF-1 as compared to that was with IGF-1 or IGFBPL1 alone. The western blot images were cropped for better overview. The original images are shown in Supplementary Fig. 4. (**E**) Representative photomicrographs of cultured RGCs immunolabeled for phospho-PI3K (P-PI3K) or phospho-mTOR (P-mTOR) after being treated with IGFBPL1 + IGF-1, IGFBPL1 + IGF-1 + BAPTA or FPL64176. Scale bar: 20 um. (**F**) Quantification of fluorescent intensity of P-PI3K in control and different treatment groups. BAPTA inhibited IGFBPL-1 and IGF-1 induced phosphorylation of PI3K, while administration of FPL64176 alone was sufficient to induce robust PI3K phosphorylation to an extent that was comparable to IGFBPL1 and IGF-1 co-treatment. ***P < 0.001, **P < 0.01, *P < 0.05, as indicated by two-tailed student *t*-test. Error bars indicate standard error of mean (SEM).

As Phosphoinositide 3-kinase/mammalian target of rapamycin (PI3K/mTOR) are indicators of axon growth capacity and important downstream signals of RGC axon growth cascades^22^, we examined if co-application of IGFBPL1 and IGF-1 or elevation of [Ca^2+^]_i_ activates these signals. Western blot analysis showed that neither IGF-1 nor IGFBPL1 alone induced PI3K phosphorylation in P10 RGCs when compared to vehicle-treated controls (Fig. 8A–D). In contrast, co-administration of IGF-1 and IGFBPL1 induced a 6-fold increase in PI3K phosphorylation and a 3-fold increase of mTOR phosphorylation compared to the control group (Fig. 8A–D, Supplementary Fig. 5). Phosphorylation of PI3K and mTOR induced by IGFBPL1 and IGF-1 co-administration was completely blocked by Ca^2+^ chelator BAPTA (1,2-bis(o-amino phenoxy)ethane-N,N,N′,N′-tetraacetic acid). Administration of L-type Ca^2+^ channel activator FPL, in contrast, resulted in robust PI3K and mTOR activation, similar to that seen in cultures receiving IGFBPL1 and IGF-1 co-treatment (Fig. 8E,F). Together, our data establishes that IGFBPL1 functions as a co-factor of IGF-1 to enable axonal growth via signaling intracellular Ca^2+^-mediated events and activating PI3K and mTOR.

## Discussion

The present study has identified a novel regulator and IGF-1 co-factor, IGFBPL1, in the mediation of RGC axon growth *in vitro* and *in vivo*. IGFBPL1 deficiency impaired axon growth, while administration of IGFBPL1 promoted RGC axonal growth in the presence of IGF-1. We demonstrate that presence of IGFBPL1 enables IGF-1 to activate intracellular Ca^2+^ signaling and PI3K and mTOR pathways and stimulate axon growth. The study unveils a new regulatory element in the pleiotropic functions of IGF-1.

During neural development, IGF-1 regulates many, and sometimes dichotomous, aspects of intracellular signals that support neural survival, differentiation, proliferation, axon growth, and synaptogenesis^23^. It is evident that IGF-1 regulates the initiation of axon growth and specification during development^24,25^; whereas, it promotes only neuronal survival without stimulating axon growth or regeneration in the *adult* CNS^24,26–28^. The molecular mechanisms underlying the shift of the pleiotropic activities of IGF-1 at various neuron developmental stages are unknown. Our study suggests that IGFBPL1, which is transiently expressed during the period of exuberant axon growth in development, is an essential co-factor of IGF-1 to enable the axon growth signaling. Restrained IGFBPL1 expression during distinct stages of RGC development thus may set the timing for RGC axon elongation. Up to date, little is reported regarding the transcriptional regulation of IGFBPL1 expression. There is much to be discovered about the functional roles for IGFBPL1 and to elucidate if IGFBPL1 also acts independent of IGF-1 to drive other cellular functions during development and response to neural injury or stress. In any case, these studies define a previously unknown signaling loop in IGF-1 function and reveal a novel mechanism that switches off the axonal growth activity of IGF-1.

Our results showed that IGFBPL1 functions through interacting with IGF-1 to enable intracellular Ca^2+^ signaling and initiate the axon growth program, such as the phosphorylation of PI3K and mTOR. IGF-1 mediates Ca^2+^ signaling pathways via activation of voltage-dependent Ca^2+^ channels and Akt phosphorylation^29,30^. Ca^2+^ as a second messenger plays pivotal roles in a variety of cellular processes in neurons, ranging from gene expression, neurite growth, synaptogenesis, to neurotransmitter release^31^. Excessive intracellular Ca^2+^ can also signal biochemical pathways initiating inflammation, free radical generation, and apoptosis^32^. Subcellular localization or transfer of Ca^2+^ from the endoplasmic reticulum (ER) to the mitochondria is important in the control of pro-survival/pro-death pathways. We previously reported that when injury-induced influx of Ca^2+^ is taken up by the mitochondria, it initiates apoptosis. Blockade of Ca^2+^ uptake by the ER and mitochondria via overexpressing Bcl-2 results in elevation of intracellular Ca^2+^ that in turn triggers signaling cascades of CREB (cAMP response element-binding protein) and Erk (Extracellular receptor kinase) and elicits neurite outgrowth and axon regeneration^8^. During development, elevated levels of intracellular Ca^2+^ ^8,33^ and mTOR phosphorylation^22^ are distinguishing features of neurons which actively extend axons^34,35^. These dynamics of intracellular Ca^2+^ level change and mTOR activation correlate with the expression of IGFBPL1 in the developing retina, supporting a role for IGFBPL1 in this regulation. Studies have shown that IGFBPL1 is widely localized in the developing mouse brain with a spatiotemporal pattern closely coincides with the time window when neurons are extending axons, but restricted to late-born neurons in the postnatal stage^36^. These observations suggest a conserved mechanism which may involve IGFBPL1 in controlling axonal growth in other CNS regions.

The relatively mild impact of gene deletion of IGFBPL1 on RGC axon growth *in vivo* implicates functional redundancy or existence of parallel pathways in the mediation of IGF-1 signaling. To date, at least 7 IGFBPs have been identified, and the involvement of these proteins in axon growth have not been well characterized. Our screening for other IGFBP members during the period of exuberant RGC axon growth detected dynamic expression of other IGFBPs in the developing retina. It remains possible that other IGFBP family members may carry out parallel functional roles as IGFBPL1 to mediate the axon growth signaling of IGF-1. Moreover, many transcription factors, including p53, SnoN, E47, CREB, STAT3, NFAT, c-Jun, ATF3, Sox4, Sox11, NFκB, PTEN, KLFs, *etc*., have been shown to regulate neurite growth *in vitro* and *in vivo*^37–39^. Screening of these transcription factors in *Igfbpl1*^−/−^ mice suggests that IGFBPL1 critically regulates ATF3 and KLF4 expression in developing RGCs (unpublished data). Uncovering the specific subsets of transcription factors underlying the secretory factor IGFBPL1 or hormonal control of axon growth is not only of potential therapeutic importance, but would also further our understanding for the mechanisms of nerve regeneration. In the future, it would be most interesting also to elucidate how IGFBPL1/IGF-1 signaling participates in the regulation of axon development and if other IGFBPs play an agonistic or antagonistic role in IGF-1-mediated axon growth activities.

In summary, the present study has revealed a novel secretory factor that participates in the activation of the axon growth machinery in developing RGCs through an IGF-1-dependent mechanism. These findings offer new perspectives for contemplating axon development regulation and regeneration and provide additional guidance in developing new therapeutic approaches to treat optic nerve injury and degeneration.

## Materials and Methods

### Mice

C57BL/6 J wild-type (Charles River Laboratories) and *Igfbpl1*^−/−^ mice at a C57BL/6 J genetic background (Knockout Mouse Project Repository, University of California at Davis) were used in these experiments. All mouse studies were approved by the Schepens Eye Research Institute Animal Care and Use Committee and performed in accordance with institutional and federal guidelines.

### Immunohistochemistry

Retinal sections (10 μm thickness) and retinal whole-mounts were prepared, and immunofluorescent labeling was performed as previously described^40^. Retinal whole-mounts were incubated with primary antibody for 24 hours at 4 °C, followed by three washes in 0.01 M Phosphate buffered saline and incubation with secondary antibody for 2 hours at room temperature. Retinal whole-mounts or sections were then mounted with VECTORSHIELD mounting medium containing DAPI (Vector Laboratories). Primary antibodies against Tuj1 (Millipore, 1:800), IGF-1 (R&D systems, 1:50), and IGFBPL1 (R&D systems, 1:50) were used. As antibody specificity or negative controls for IGF-1 and IGFBPL1, the primary antibodies were omitted from the staining procedure, and P0 retinal sections (located in the same sections) which stain specifically with the primary antibody were used as positive controls. The specimens were visualized and photographed under Leica confocal microscope.

For RGC counting, retinal flat-mounts were divided into quadrants using the optic nerve head (ONH) as the origin: superior, temporal, nasal and inferior. Within each quadrant, four squares (198 µm × 198 µm) distributed at a 1 mm interval along the radius were selected: one from the peripheral region (2 mm from the ONH), two from the intermediate region (1 mm from the ONH), and one from the central region. Thus, total 16 square regions of each eye were photographed, and all Tuj1^+^ cells in the ganglion cell layer were counted. Average RGC densities of the entire retina were calculated.

Immunocytochemistry was used to determine RGC cell survival, axonal growth, and activation of signaling molecules including PI3K and mTOR. Briefly, cultured RGCs in 96-well plate were fixed with 2% paraformaldehyde for 15 min followed by immunostaining with primary antibodies against RGC marker Tuj1 (Millipore, 1:800), Phospho-PI3K (Cell Signaling, 1:50), or Phospho-mTOR (Cell Signaling, 1:50). Corresponding secondary antibodies were obtained from Jackson ImmunoResearch Laboratories Inc. Confocal images were acquired using a Leica confocal microscope and the LAS AF software. At least 8 images were acquired from each well. The longest axon of each cell, the number of cells bearing axons, and the fluorescent intensity of phosphor-PI3K or phosphor-mTOR were measured using ImageJ. All assays were repeated at least 4–6 times, and quantifications were carried out in a double-blind fashion.

### Quantitative RT-PCR

RNA was prepared from freshly dissected retinas and cDNA was synthesized using the SuperScript^®^ III First-Strand Synthesis System (Life Technologies). Quantitative PCR was performed using KAPA SYBR^®^ FAST 2× qPCR Master Mix (Kapa Biosystems) and Eppendorf Mastercycler ep realplex^2^ (Eppendorf North America). Primers used in this study: IGFBPL1 Forward 5′ CTGTATGACCCTGGGCAAGT 3′; Reverse 5′ GCCAGACCCAATTACGTGTT 3′; GAPDH Forward 5′ AACTTTGGCATTGTGGAAGG 3′; Reverse 5′ TGTTCCTACCCCCAATGTGT 3′.

### Neuron culture preparation and quantification for cell survival and neurite growth

Purification of RGCs was performed as previously described^41^. RGCs (5 × 10^4^ cells/well) were seeded in 96-well culture plates that were pre-coated with Poly-D-Lysine (Millipore, 0.1 mg/ml) and merosin (EMD Millipore, 5 μg/ml), and maintained in a humidified tissue culture incubator in the presence of 5% CO_2_ for 3 days. In some experiments, the following reagents or chemicals were added into the culture medium immediately after the cells were seeded: recombinant mouse IGF-1 (R&D systems), recombinant mouse IGFBPL1 (R&D systems), H-1356^24,42^ (Bachem, 40 μM), and NBI-31772^14^ (EMD Millipore, #479830, 10 μM). After 3 days of incubation, cells were washed with Dulbecco’s Phosphate-Buffered Saline and stained with CalceinAM and EthD-1 (LIVE/DEAD^®^ Viability/Cytotoxicity kit, Life Technologies) for 30 min at room temperature. Five images, one from the center and four from the periphery, were obtained from each well using an Olympus inverted fluorescence microscope. Live and dead cells were counted using ImageJ software, and the rates of cell survival were calculated as *Live cells/(Live* + *Dead cells)%*. To quantify for axonal outgrowth, cells were fixed with 2% paraformaldehyde for 15 min followed by immunostaining with primary antibody against RGC marker Tuj1 (1:800) and corresponding secondary antibody immunostaining. The longest axon of each cell and number of cells bearing axons were measured using ImageJ. All assays were repeated in at least 4–6 independent experiments, and quantifications for cell survival and axonal growth were carried out in a masked fashion.

PC12 cells were cultured in RPMI supplemented with 5% FBS, 10% horse serum, and a mixture of 1% of penicillin/streptomycin and incubated at 37 °C in a humid 5% CO2 environment. PC12 cells were differentiated with 100 ng/ml nerve growth factor for 5 days under different conditions. For hippocampal neuron cultures, hippocampi were dissected from P0 mouse pups, dissociated with papain (Worthington Biochemical, LK003150) and incubated as described above in a humidified tissue culture incubator with 5% CO2 for 3 days.

### Acute knockdown of IGFBPL1 and IGF-1 with lentiviral shRNA

IGFBPL1 shRNA and scrambled shRNA (Sigma-Aldrich) were packaged individually into a lentiviral vector (pLKO.1-puro-CMV-TurboGFP^TM^-igfbpl1 and pLKO.1-puro-CMV-TurboGFP^TM^-scrambled shRNA) by DOM Vector Core at the University of California at Los Angeles. Transduction was carried out in 96-well culture plates with 0.5 μL of lentivirus stock solution (~1 × 10^8^ TU/ml) in each well that were pre-seeded with 5 × 10^4^ primary RGCs for 6 hours. Cells were allowed to grow for 3 days before analysis.

### Co-IP and Western blots

Co-immunoprecipitation (co-IP) of IGFBPL1 and IGF-1 was performed using the Pierce Co-IP kit (Thermo Scientific) following manufacturer’s instruction. Briefly, 50 μl of resin was loaded into the Pierce Spin Column and washed twice with 200 μL of 1× Coupling Buffer. IGFBPL1 antibody (16 μg; goat anti-IGFBPL1, R&D systems) or IGF-1 antibody (goat anti-IGF-1, R&D systems) was diluted in 200 μL of 1× Coupling Buffer containing 3 μL of the Sodium Cyanoborohydride Solution and was incubated with resin in the spin column for 120 min at room temperature on a mini rotator. The spin column was washed twice with 200 μl of 1× Coupling Buffer, followed by a wash with quenching buffer, and incubated with 200 μL of quenching buffer containing 3 μL of the Sodium Cyanoborohydride Solution for 15 min with gentle shaking. The resin was washed with 200 μL of 1× Coupling Buffer twice followed by 6 washes with 150 μl of Wash Solution. Retinal lysate from P0 mouse pups was pre-cleared using the control agarose resin. The antibody-coupled resin was washed with 200 μl of IP Lysis/Wash Buffer twice, and 200 μl of pre-cleared retinal lysate was added and incubated at 4 °C overnight. The resin column was washed with 200 μl of IP Lysis/Wash Buffer three times and centrifuged to remove the Lysis/Wash Buffer. Captured proteins were eluted by 50 μl of elution buffer and were examined for the presence of IGFBPL1 or IGF-1 by Western Blot. Resin was regenerated and stored for future use. Western blot was performed as previously described^43^. In brief, the retinas were homogenized, and 30–50 μg of total protein from retina homogenates were run on a 4–20% polyacrylamide gel. Membranes were blocked with 25 mM Tris·Cl (pH 7.5) buffer containing 5% non-fat milk, and probed with appropriate primary and secondary antibodies. The IRDy^®^-conjugated secondary antibodies were visualized with the LI-COR Odyssey^®^ system, and the horseradish peroxidase (HRP)-conjugated secondary antibodies (Pierce, Rockford, IL). Signals were detected with enhanced SuperSignal West Chemiluminescent Substrates on CL-XPosure Film (Thermo Scientific).

### Measurement of [Ca^2+^]_i_

RGCs were cultured for 48 hours in multi-chamber culture slides pre-coated with Poly-D-Lysine and merosine. Cells were loaded with 1 μM Fura-2 and 8 μM pluronic acid F127 in the culture medium at 37 °C tissue culture incubator for 30 min. Cells were then washed twice with mammalian Ringer’s solution and maintained in Ringer’s solution containing 250 μM sulfinpyrazone for calcium imaging. Recombinant mouse IGF-1 and IGFBPL1 were dissolved in Ringer’s solution containing 250 μM sulfinpyrazone. Real-time imaging of intracellular calcium was acquired using a ratio imaging system InCyt Im2 (Intracellular Imaging, Cincinnati, OH) at the excimer wavelengths of 340 and 380 nm and an emission wavelength of 505 nm. Data was presented as the actual [Ca^2+^]_i_ with time.

### Treatment of cultures with calcium modulators

RGCs were purified and cultured with the presence or absence of IGFBPL1 + IGF-1 and chemicals that modulate intracellular calcium levels for three days. The calcium chelators EGTA (0.1 mM) and the calcium channel blocker Nifedipine (25 μM) were used to lower intracellular calcium concentration [Ca^2+^]_i_; the L-type calcium channel activator FPL-64176 (0.5 μM) was used to increase [Ca^2+^]_i_. After three days of treatment, cells were loaded with Calcein AM for axonal growth measurements.

### Axon counts in optic nerve cross sections

Mouse optic nerve samples were fixed with half-strength Karnovsky’s fixative. Semi-thin optic nerve cross sections (1.0 μm) taken at 2 mm posterior to the globe were stained with 2% paraphenylenediamine aqueous solution to stain the axons. Twelve square regions (22.05 µm × 20.64 µm) distributed at four quadrants, of which 4 were taken from the central region and 8 from the peripheral region, were photographed. Using ImageJ, all axons in the photomicrographs were automatically counted and the areas of the optic nerve sections were measured. Axonal density was recorded, and the total axon number per optic nerve was calculated as (axon density x total area of the nerve cross section). All quantification procedure was carried out in a masked fashion.

## Electronic supplementary material

Supplementary Figures
