# Supplementary figures and images for: IGFBPL1 Regulates Axon Growth through IGF-1-mediated Signaling Cascades
Source: Sci Rep. 2018 Feb 1;8:2054. doi: 10.1038/s41598-018-20463-5 (PMC5794803; doi:10.1038/s41598-018-20463-5)

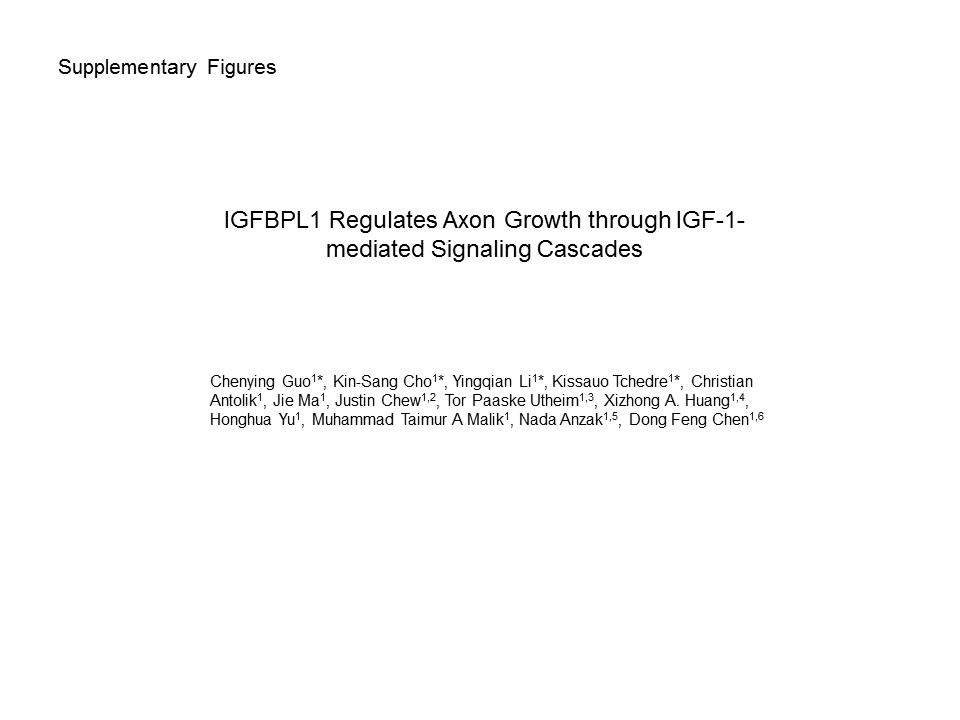

Supplement: Supplementary file 1 — Supplementary Figures [file 41598_2018_20463_MOESM1_ESM.tif]
